# Mortality and risk factors associated with pulmonary embolism in coronavirus disease 2019 patients: a systematic review and meta-analysis

**DOI:** 10.1038/s41598-021-95512-7

**Published:** 2021-08-06

**Authors:** Carlos Andrés Gómez, Cheuk-Kwan Sun, I-Ting Tsai, Yang-Pei Chang, Ming-Chung Lin, I-Yin Hung, Ying-Jen Chang, Li-Kai Wang, Yao-Tsung Lin, Kuo-Chuan Hung

**Affiliations:** 1 School of Medicine for International Students, College of Medicine, I-Shou University, Kaohsiung, Taiwan; 2 Universidad Nacional Autónoma de Honduras en el Valle de Sula, San Pedro Sula, Honduras; 3 Department of Emergency Medicine, E-Da Hospital, Kaohsiung, Taiwan; 4 Department of Neurology, Kaohsiung Municipal Ta-Tung Hospital, Kaohsiung Medical University, Kaohsiung, Taiwan; 5 Department of Neurology, Kaohsiung Medical University Hospital, Kaohsiung Medical University, Kaohsiung, Taiwan; 6 Department of Anesthesiology, Chi Mei Medical Center, No. 901, ChungHwa Road, YungKung Dist, Tainan, 71004 Taiwan

**Keywords:** Medical research, Risk factors

## Abstract

To determine, in patients with coronavirus disease 2019 (COVID-19) infection, the associations of pulmonary embolism (PE) with mortality and risk factors for PE as well as the therapeutic benefit of anticoagulant prophylaxis. Embase, PubMed, Cochrane controlled trials register, and Web of Science databases were searched from inception to October 10, 2020. We included all published trials on PE in patients diagnosed with COVID-19 with eligibility of the trials assessed following the PRISMA guidelines. Sixteen clinical trials with 5826 patients were eligible. There were significant associations of PE with the male gender [odd ratio (OR) = 1.59, 95% CI 1.28–1.97], mechanical ventilation (OR = 3.71, 95% CI 2.57–5.36), intensive care unit admission (OR = 2.99, 95% CI 2.11–4.23), circulating D-dimer [mean difference (MD) = 5.04 µg/mL, 95% CI 3.67–6.42) and CRP (MD = 1.97 mg/dL, 95% CI 0.58– 3.35) concentrations without significant correlation between PE and mortality (OR = 1.31, 95% CI 0.82–2.08) as well as other parameters or comorbidities. After omitting one trial with strict patient selection criteria for anticoagulant prophylaxis, significant prophylactic benefit was noted (OR = 0.31, 95% CI 0.1–0.91). Our findings identified the risk factors associated with PE in COVID-19 patients and supported the therapeutic benefit of anticoagulant prophylaxis against PE in this patient population.

## Introduction

Venous thromboembolism represents the third most common vascular disease after acute myocardial infarction and stroke^1^. Accumulating evidence has shown an increased risk of thrombotic complications in patients with coronavirus disease 2019 (COVID-19)^2^ whose prevalence of thromboembolism is up to 20–25%^2,3^ compared with a lifetime risk of 8% in the general population^4^. Indeed, the figure may be an underestimate taking into account the postmortem finding that over 50% of COVID-19 patients may have undetected thromboembolism before demise^5^. A previous study reported an increased risk of thromboembolic complications in patients with severe COVID-19 infection, particularly those admitted to the intensive care unit (ICU)^2^. In concert with this finding, patients with severe COVID-19 could experience a 3.76-fold elevation in risk for thromboembolism compared to that in those with a non-severe disease according to a previous meta-analysis^3^. The risk of thrombosis was also considered high in COVID-19 patients with obesity and acute respiratory distress syndrome as well as those undergoing extracorporeal membrane oxygenation (ECMO) and those with hypercoagulability (e.g., fibrinogen > 8 g/L and/or D-dimers > 3 μg/mL) and/or marked inflammatory syndrome^6^.

Prognostically, thromboembolism is believed to contribute to mortality and morbidity in patients infected with COVID-19^3,7,8^. Pulmonary embolism (PE) and deep vein thrombosis (DVT) are the two COVID-19-related thromboembolic complications with the prevalence of the former being two-fold higher than that of the latter.^3^ A small autopsy series identified PE as the cause of death in up to one-third of patients with COVID-19 (i.e., 4 out of 12)^5^.

Regarding the mechanism underlying the hypercoagulable state in patients with COVID-19, previous studies have identified two distinct phenotypes of thrombotic manifestations. In addition to thromboembosis^9,10^ that is also observed in other septic situations, COVID-19 is characterized by another micro-thrombotic pattern prevailing in the lungs resulting from a massive coagulation activation accompanied by intense inflammatory and immune reactions^11,12^. The latter, which is termed "immuno-thrombosis"^12^, could cause widespread occlusive thrombotic micro-angiopathy and destruction of alveoli^11^. Pathologically, entering of SARS-CoV-2 into the airway epithelial cells triggers a cascade of inflammatory and immune reactions, including alveolar infiltrations of macrophages, monocytes, and T cells as well as the generation of chemokines and cytokines including TNF-α, IL-1β, IL-6, and IL-8, leading to an elevated fibrin degradation and an increase in D-dimer levels^12^. A previous clinical investigation has shown a persistent elevation in D-dimer levels (> 500 ng/mL) in patients with COVID-19 up to four months after their convalescence despite the normalization of other coagulation and inflammation markers^13^, highlighting the immunological nature of COVID-19-related hypercoagulation.

Accordingly, anticoagulant prophylaxis against thrombosis, which commonly involves the use of unfractionated heparin or low molecular weight heparin^7,14^, has become a standard treatment protocol for patients with confirmed diagnosis of COVID-19^6^.

Nevertheless, despite standard anticoagulant thromboprophylaxis, the incidence of COVID-19-related thrombosis remains high^15,16^ at least partly attributable to heparin resistance^14^. Because of the limitations in previous clinical trials on thromboprophylactic strategies, a collaborative effort has been proposed to conduct pooled analyses and expedite the implementation of effective interventions^15^. Indeed, the risk factors for PE, the impact of PE on mortality, and the effectiveness of anticoagulant prophylaxis against PE in patients with COVID-19 infection remain pressing issues that have not been systematically addressed. Therefore, through analysing available data from eligible trials, the present systematic review and meta-analysis aimed at investigating the association of PE with mortality, identifying the risk factors for PE as well as assessing the therapeutic benefit of anticoagulant prophylaxis in patients infected with COVID-19.

## Methods

### Protocol registration

We registered the protocol of the current study with PROSPERO (CRD42020213355).

### Search strategy

We conducted the present meta-analysis according to Preferred Reporting Items Systematic Reviews and Meta-Analysis (PRISMA) guidelines^17^. We searched the databases of Embase, PubMed, Cochrane controlled trials register, and Web of Science to obtain a list of all published eligible trials using the keywords "thromboembolism", "clot", "deep vein thrombosis (DVT)", "venous thromboembolism", "pulmonary embolism", "thrombosis", "venous thrombosis", "severe acute respiratory syndrome", "coronavirus 2", "coronavirus", "corona virus", "covid-19", "nCoV", "2019nCoV" or "Wuhan virus" from inception to October 10, 2020. References from relevant studies were searched to find additional articles. No publication date or language restriction was applied.

### Study selection criteria

Two reviewers independently examined the titles and abstracts of the articles to identify potentially eligible studies. The inclusion criteria for eligibility of trials for the current study included studies which compared the patient characteristics, laboratory profiles, and outcomes in COVID-19 patients with or without the occurrence of PE. The exclusion criteria were (1) studies that focused on patients with pregnancy, pediatric population, patients receiving extracorporeal membrane oxygenation (ECMO) as well as those with immune diseases (e.g., rheumatic arthritis); (2) those whose information regarding outcomes (e.g., patient characteristics) was unavailable; (3) postmortem studies; (4) case series; and (5) those with mixed outcomes from patients with DVT and PE without distinct information from patients with PE. Two authors independently investigated the selected studies for the final analysis. In the situation of disagreements, a third author was involved until a consensus was reached. Kappa statistics for interrater agreement evaluation (Moderate: 0.41–0.60; Substantial: 0.61–0.80; Almost perfect: 0.81–1.00)^18^ were used to assess the degree of agreement between the two reviewers.

### Data extraction

Two authors were responsible for extracting relevant data from each selected trial and entering them into predefined databases. Divergences were resolved through discussion. The corresponding authors of the included studies that did not provide data on primary or secondary outcomes were contacted for further information. The data extracted from each trial were as follows: year of publication, author, study setting (e.g., retrospective design), sample size, patient characteristics (e.g., gender), body mass index (BMI), the use of anticoagulant prophylaxis, laboratory profiles (e.g., D-dimer), and outcomes (e.g., mortality).

### Primary outcome, secondary outcomes, and definitions

The primary endpoint was the risk of mortality, while the secondary outcomes were potential risk factors for PE and changes in laboratory profiles with or without the occurrence of PE during the study period. If the same laboratory parameter was available at different time points, only the maximum value was selected for analysis.

### Assessment of risks of bias for the included studies

Two authors assessed the risks of bias of the included non-randomised studies with the Newcastle-Ottawa Scale (NOS) for quality of cohort studies^19^, which scores each study based on three domains, namely, study group selection, group comparability, and outcome of interest ascertainment for cohort studies. A maximum of four, three, and two stars were assigned to the Selection, Comparability, and Outcome domains, respectively. The higher the number of stars, the better the quality of the study (i.e., up to nine stars for highest quality studies)^19^. For the second item of the Outcome domain, we awarded one star to a study if the patients were discharged from hospital or succumbed to the disease. For non-hospitalised patients or lack of relevant information (e.g., discharge or fatality) for inpatients, no star was assigned with the assumption of inadequate follow-up. One star was given to the last item of the Outcome domain for (1) studies with a follow-up rate ≥ 80%, or (2) those with a follow-up rate < 80% but including a description of lack of significant difference in demographic characteristics between the follow-up and lost-to-follow-up groups. Studies with less than six stars were considered to be of low-quality.

### Statistical analysis

For dichotomous outcomes, a random effects model was used to calculate the odd ratios (ORs) with 95% confidence intervals (CIs). The Mantel–Haenszel (MH) method was used to pool dichotomous data and to compute pooled ORs with 95% CIs. For continuous outcome, the selected effect size was expressed as mean difference (MD). The I^2^ statistics was adopted to assess the heterogeneity, which was categorised as low (0–50%); moderate (51–75%), and high (76–100%). Sensitivity analyses were performed to evaluate the potential influence of a single trial on the overall results by removing the studies from the meta-analysis one at a time. In addition, to refine the quality of the present meta-analysis, we re-assessed the overall results after removing the low-quality studies. Funnel plots were used for investigating the potentials of reporting and publication bias when a particular outcome was reported in 10 or more studies. Statistical significance was set at 0.05 for all analyses. Cochrane Review Manager (RevMan 5.4; Copenhagen: The Nordic Cochrane Center, The Cochrane Collaboration, 2014) and MetaXL (version 5.1) was used for data synthesis. Statistical Product and Service Solutions (SPSS, version 22.0; Chicago, IL) was used for the calculation of kappa coefficient.

## Results

### Study selection

**Figure 1:**
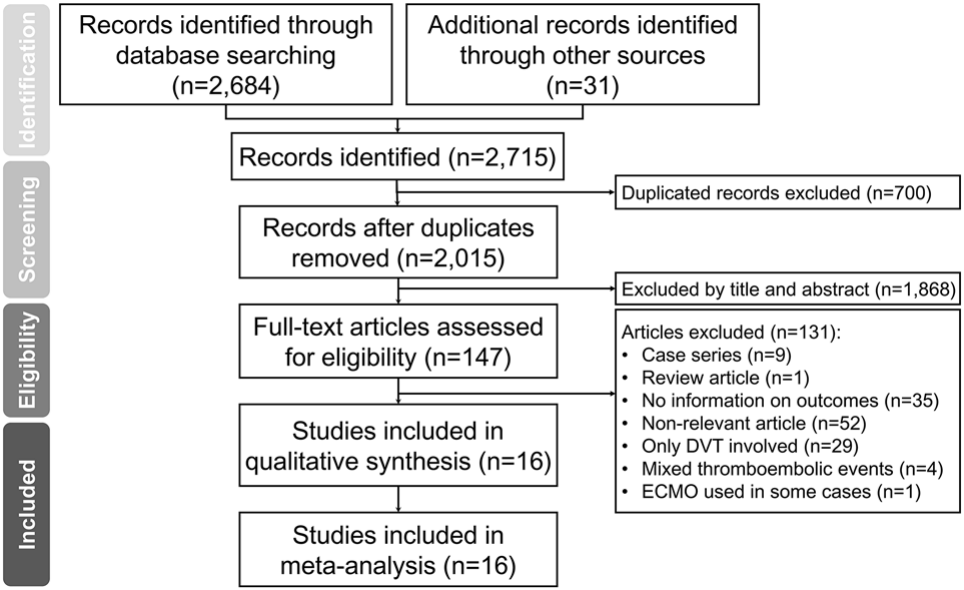
PRISMA flowchart for selecting eligible studies. DVT = deep vein thrombosis, ECMO = extracorporeal membrane oxygenation.

Figure 1 is the Preferred Reporting Items for Systematic reviews and Meta-Analyses (PRISMA) flow diagram that summarises the reasons for study exclusion. Of a total of 2715 potentially eligible studies retrieved from the database search, 700 were removed because of duplication. We then excluded 1868 records after the initial review of the titles and abstracts. Of the remaining 147 articles for full-text review, 131 were excluded because of their natures of case series (n = 9) or review article (n = 1), no information on outcomes (n = 35), content not related to the present study (n = 52), recruitment of only DVT patients (n = 29), mixed thromboembolic events (n = 4), and the use of extracorporeal membrane oxygenation (ECMO) in some patients (n = 1). Finally, a total of 16 studies with 5826 patients were included in the current meta-analysis^20–34^ (Fig. 1). There was a high inter-observer reliability in article selection (kappa = 0.82).

### Characteristics of included studies

**Figure 2:**
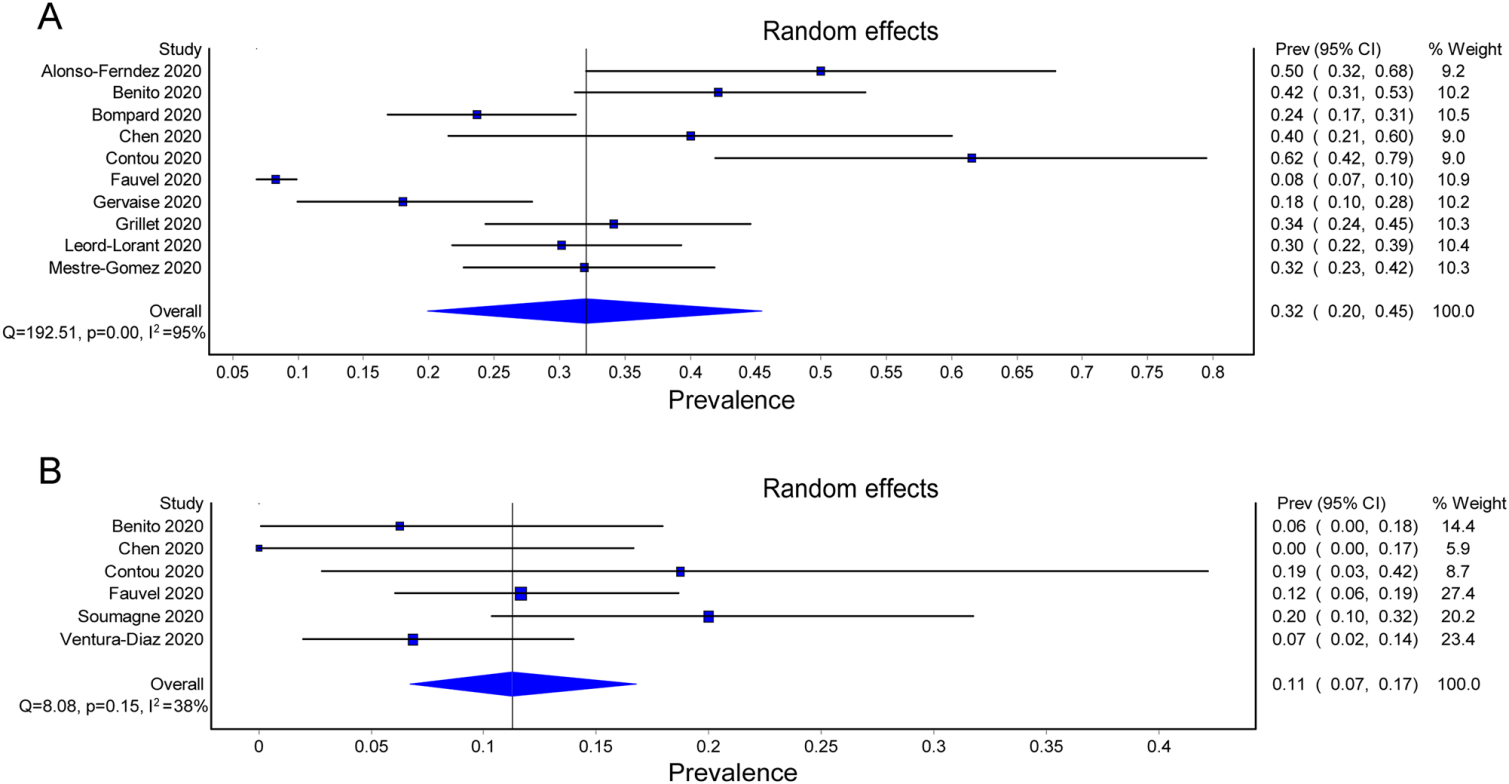
(**A**) The prevalence of pulmonary embolism in COVID-19 patients receiving computerized tomography pulmonary angiography (n = 10). (**B**) Reports on concurrent deep vein thrombosis (DVT) in COVID-19 patients with pulmonary embolism (n = 8).

The study characteristics are described in Table 1. The countries of origin of the 16 studies were France (n = 7), Spain (n = 4), France and Belgium (n = 1), Belgium (n = 1), China (n = 1), United States (n = 1), and the United Kingdom (n = 1). The study design was prospective in three studies ^20,21,31^ and retrospective in the other thirteen studies^22–34^. The sample size ranged from 25 to 2907 with a male predominance (58.1%–84.6%). The study populations included hospitalised adult patients (e.g., those in the intensive care unit [ICU] and in wards) (number of studies = 11)^20–22,24,26,28–31,33,34^, both inpatients and outpatients (number of study = 1)^28^, non-hospitalised patients (e.g., those visiting the emergency department) (number of study = 1)^27^, and only ICU patients^25,31,32^ (number of study = 3). Computed tomography is the gold standard for the diagnosis of PE^35^. Although most studies described the indications for computed tomography pulmonary angiography (CTPA)^20–22,24–31,33,34^ (Supplemental Table 1), three did not specify such indications^23,31,32^. The prevalence of PE in patients receiving CTPA screening ranged from 8.3 to 61.5%, with a pooled prevalence of 32% (95% CI 19.9–45.5%) (Fig. 2A). Although most studies described anticoagulant prophylaxis^20–23,26,29–32,34^, five did not specify such strategies^24,25,27,28,33^. Eight studies, which reported concurrent DVT in PE patients (Supplemental Table 2)^21,24–26,31–34^, showed a pooled DVT prevalence of 11% in this patient population (95% CI 7–17%) (Fig. 2B).

**Table 1:** Characteristics of the included studies (n = 16).

Study	Study Design	Hospital/Country	Total Patients	Age (year)	Male (%)	Prevalence of PE n (%)†	Anticoagulation Prophylaxis	Mortality rate (PE vs. non-PE)
Alonso-Ferndez 2020^20^	Pro	1/ Spain	30	63.9 ± 12.1	63.3	15 (50)	90%	0
Benito 2020^21^	Pro	1/ Spain	76	62.5 ± 15.6	67.1	32 (42.1)	87.5% vs. 88.6%‡	9.4% vs. 11.4%
Bilaloglu 2020^22^	Retro	4/ USA	2907	–	–	–	Low-dose (prophylaxis) anticoagulation was used in most patients	37.7% vs. 21%
Bompard 2020^23^	Retro	2/France	135	64.7 ± 17.1	70	32 (23.7)	100%	13% vs. 12%
Chen 2020^24^	Retro	1/China	25	63.8 ± 10.6	60	10 (40)	–	20% vs. 26.7%
Contou 2020^25^	Retro	1/France	26	61.7 ± 23.5	84.6	16 (61.5)	–	69% vs. 20%
Fauvel 2020^26^	Retro	24/France	1240	64.0 ± 17.0	58.1	103 (8.3)	71.4%	8.7% vs. 12.5%
Gervaise 2020^27^	Retro	1/France	72	62.3 ± 17.8	75	13 (18.1)	–	23% vs. 13%
Grillet 2020^28^	Retro	1/France	85	65.0 ± 13.0	64.7	29 (34.1)	–	–
Leonard-Lorant 2020^29^	Retro	1/France	106	63.3 ± 17.3	66	32 (30.2)	46.2%	–
Mestre-Gómez 2020^30^	Retro	1/Spain	91	65.2 ± 13.5	68.1	29 (31.9)	79.3% (23/29) of the PE patients receiving prophylactic doses	–
Mouhat 2020^31^	Retro	1/France	162	65.6 ± 13	67.3	44 (27.2)	87%	–
Soumagne 2020^35^	Pro	12/France; 9/Belgium	375	63.5 ± 10.2	77	55 (14.7)	All patients received administrated anticoagulation at least at preventive dose	29% vs. 37%
Taccone 2020^32^	Retro	1/Belgium	40	61.3 ± 6.9	70	13 (32.5)	100%	46% vs. 52%
Ventura-Díaz 2020^33^	Retro	1/Spain	242	67.0 ± 17.2	62	73 (30.2)	–	23.3% vs. 13%
Whyte 2020^34^	Retro	1/UK	214	61.1 ± 2.4	60.3	80 (37.4)	All patients received anticoagulation	–

Pro, prospective; Retro, retrospective; †Prevalence of PE in patients receiving computed tomography pulmonary angiography; USA, United States; UK, United Kingdom.

**Table 2:** Quality of included studies assessed with Newcastle Ottawa scale (n = 16).

Study	Number of stars awarded in each domain			Total score (out of 9)
	Selection (Maximum: 4★)	Comparability (Maximum: 2★)	Outcome (Maximum: 3★)	
Alonso-Ferndez 2020^20^	★★★★	★★	★★	8
Benito 2020^21^	★★★★	★★	★★	8
Bilaloglu 2020^22^	★★	★	★★	5
Bompard 2020^23^	★★	★	★★★	6
Chen 2020^24^	★★★★	★★	★★★	9
Contou 2020^25^	★★★★	★★	★★	8
Fauvel 2020^26^	★★★★	★★	★★	8
Gervaise 2020^27^	★★★★	★★	★★★	9
Grillet 2020^28^	★★	★★	★	5
Leord-Lorant 2020^29^	★★	–	★★	4
Mestre-Gómez 2020^30^	★★★★	★	★★★	8
Mouhat 2020^31^	★★★★	★★	★★★	9
Soumagne 2020^35^	★★	★★	★★	6
Taccone 2020^32^	★★	★★	★★	6
Ventura-Díaz 2020^33^	★★★★	★★	★★★	9
Whyte 2020^34^	★★★★	★	★★	7

A maximum of four, three, and two stars were assigned to the Selection, Comparability, and Outcome domains, respectively. The higher the number of stars, the better the quality of the study (i.e., up to nine stars for highest quality studies). For the second item of the Outcome domain, we awarded one star to a study if the patients were discharged from hospital or succumbed to the disease. For non-hospitalised patients or lack of relevant information (e.g., discharge or fatality) for inpatients, no star was assigned with the assumption of inadequate follow-up. One star was given to the last item of the Outcome domain for (1) studies with a follow-up rate ≥ 80%, or (2) those with a follow-up rate < 80% but including a description of lack of significant difference in demographic characteristics between the follow-up and lost-to-follow-up groups.

### Quality of included studies

Based on NOS, 81.3% (13/16) of the comparative cohort studies demonstrated an overall low risk of bias. The number of stars awarded to each of the included studies ranged from four to eight with a median NOS score of 8. Details on the numbers of stars assigned to the domains of Selection, Comparability, and Outcome for all the included studies are shown in Table 2. The most common source of bias was inadequate follow-up period in the Outcome domain^20–22,25,26,28,29,31,32,34^, followed by the lack of description on cohort derivation in the Selection domain^22,23,28,29,31,32^.

### Outcomes

#### Risk of mortality in COVID-19 patients with pulmonary embolism

**Figure 3:**
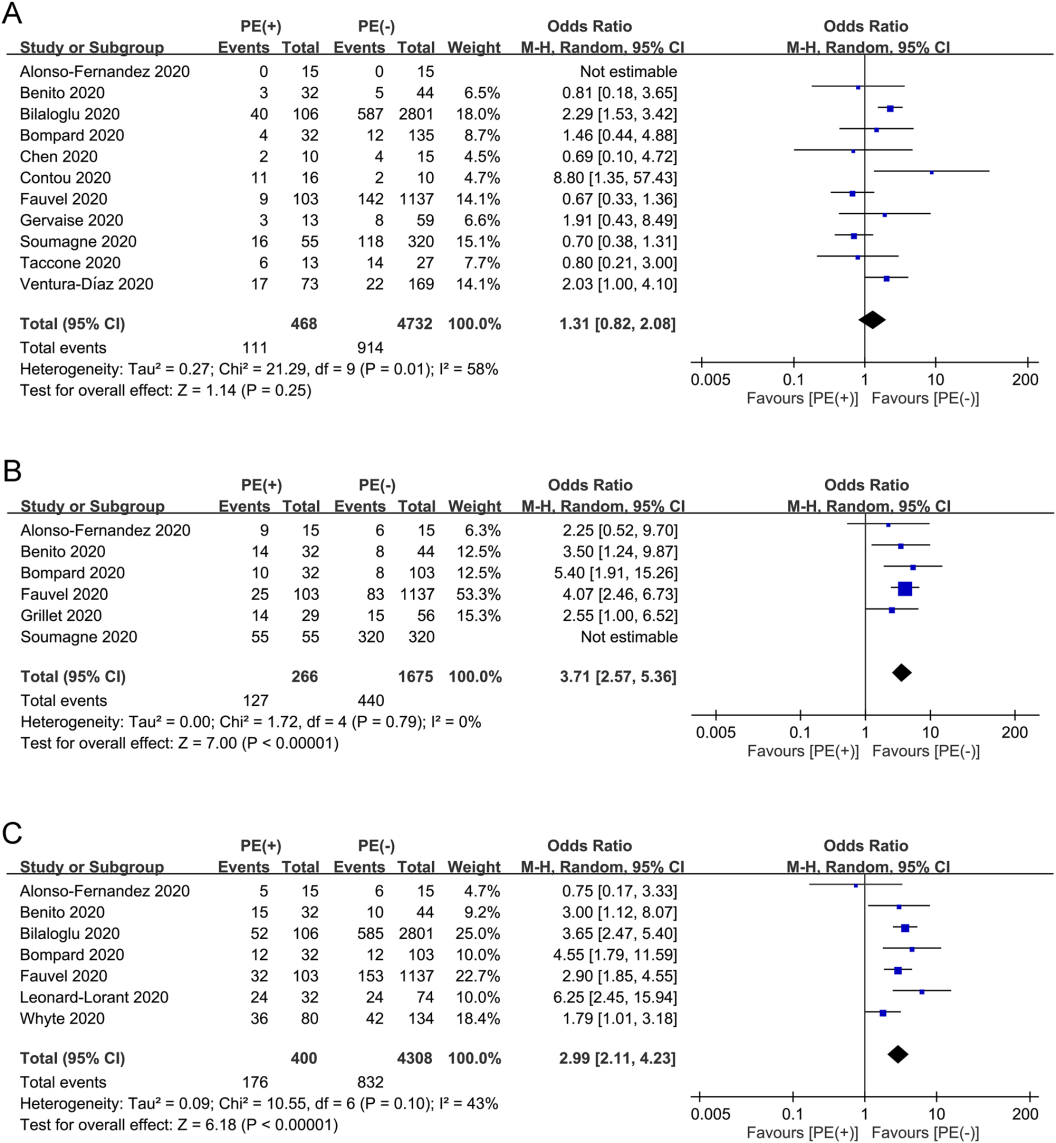
Forest plots for the comparison of risks for (**A**) mortality, (**B**) mechanical ventilation, and (**C**) intensive care unit admission between pulmonary embolism (PE) and non-PE groups. CI = confidence interval, M-H = Mantel–Haenszel.

**Figure 4:**
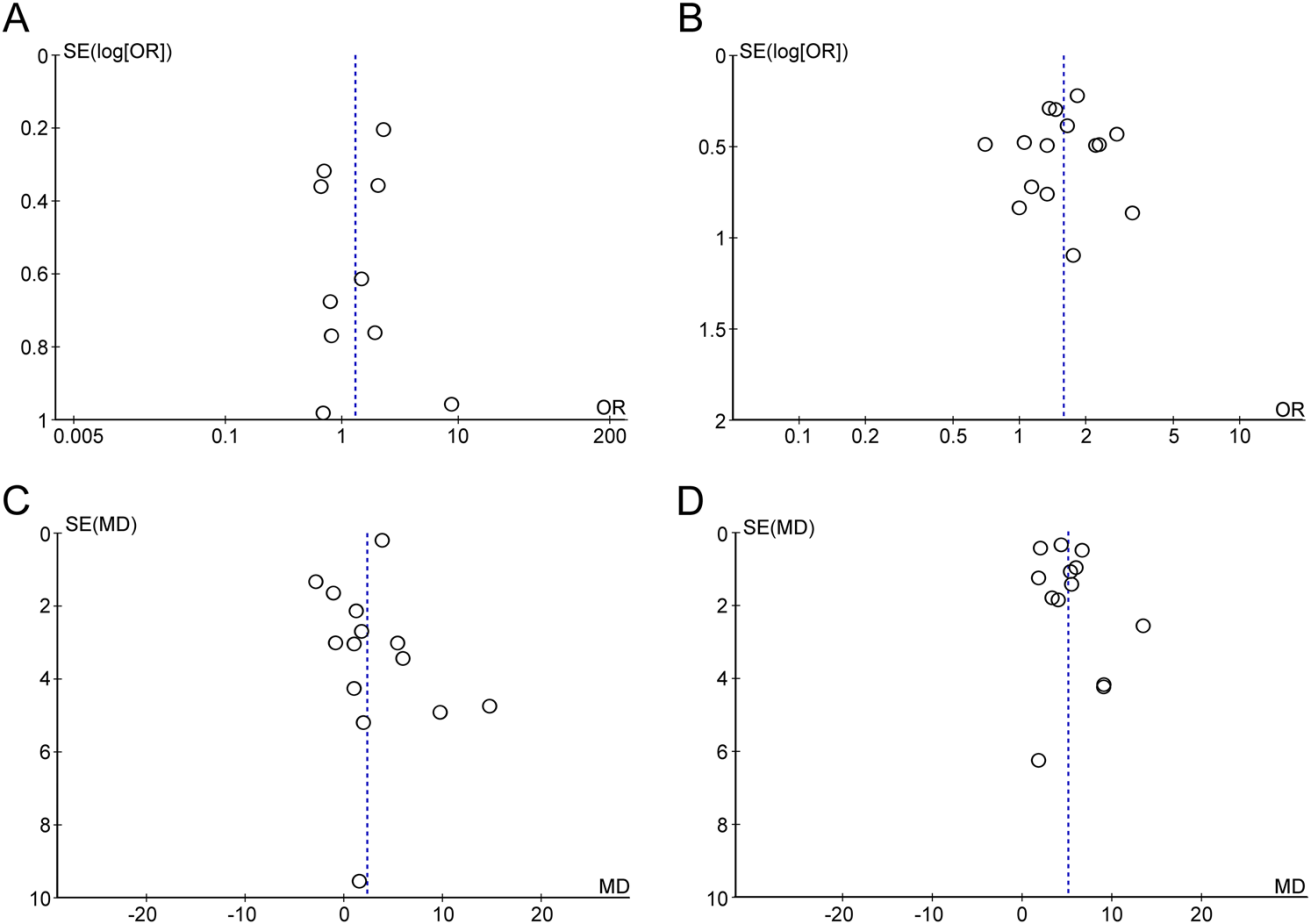
Funnel plots for estimating publication bias in studies on (**A**) Risk of mortality; (**B**) Male gender; (**C**) Age; and (**D**) Circulating D-dimer concentration. SE = standard error, OR = odds ratio, MD = mean difference.

Eleven studies with a total of 5200 patients (PE group, n = 468 vs. non-PE group, n = 4732) were available for mortality analysis^20–27,31–33^. Pooled analysis showed a comparable risk of mortality between PE and non-PE groups (OR = 1.31, 95% CI 0.82 to 2.08, *p* = 0.25; I^2^ = 58%) (Fig. 3A). Sensitivity analysis demonstrated no significant impact on outcome by omitting either one study at a time or the studies of low quality. Funnel plot demonstrated apparent symmetry (Fig. 4A), suggesting a low risk of publication bias.

#### Risk of mechanical ventilation or ICU admission in COVID-19 patients with pulmonary embolism

Six studies involving a total of 1941 patients with COVID-19 (PE group, n = 266 vs. non-PE group, n = 1675) were eligible for the analysis of the risk of mechanical ventilation^20,21,23,26,28,31^. A forest plot demonstrated a higher risk of mechanical ventilation in the PE group compared with that in the non-PE group (OR = 3.71, 95% CI 2.57 to 5.36, *p* < 0.00001; I^2^ = 0%) (Fig. 3B). Sensitivity analysis showed no significant impact on outcome by removing either one study at a time or those of low quality.

Seven studies with a total of 4708 patients (PE group, n = 400 vs. non-PE group, n = 4308) were available for assessing the impact of PE on the risk of ICU admission^20–23,26,29,34^. Forest plot analysis revealed a higher risk of ICU admission in the PE group compared to that in the non-PE group (OR = 2.99, 95% CI 2.11 to 4.23, *p* < 0.00001; I^2^ = 43%) (Fig. 3C). There was no significant impact on outcome by omitting one study at a time or removing those of low-quality.

#### Risk factors for pulmonary embolism in COVID-19 patients

**Figure 5:**
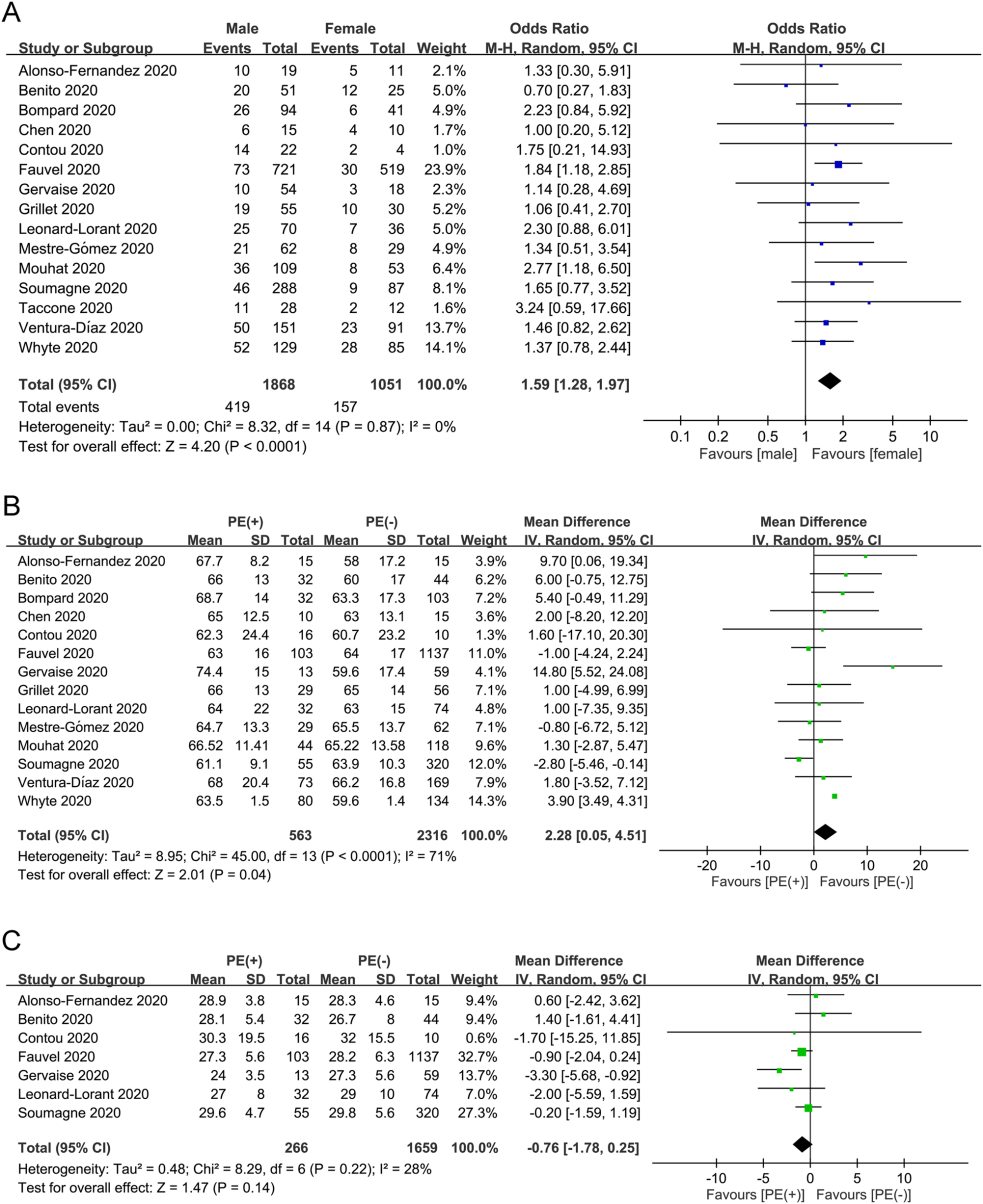
Forest plots for assessing the difference in (**A**) Gender; (**B**) Age; and (**C**) Body mass index between pulmonary embolism (PE) and non-PE groups. CI = confidence interval, M-H = Mantel–Haenszel, IV = inverse variance.

The results of the meta-analyses on studied variables including gender, age, BMI, and comorbidities are demonstrated in Fig. 5A–C and Table 3. There were 15 and 14 studies available for gender (male group, n = 1868 vs. female group, n = 1051)^20,21,23–34^ and age (PE group, n = 563 vs. non-PE group, n = 2316)^20,21,23–31,33,34^ analyses, respectively. Funnel plot exhibited apparent symmetry (Fig. 4B,C), suggesting a low risk of publication bias. Overall, male gender was a risk factor for PE (OR = 1.59, 95% CI 1.28 to 1.97, *p* < 0.0001; I^2^ = 0%) (Fig. 5A) and patients with PE were older than those without (MD = 2.28 years, 95% CI 0.05–4.51, *p* = 0.04; I^2^ = 71%) (Fig. 5B). Sensitivity analysis by omitting certain studies (i.e., one at a time or those of low quality) revealed that the male gender remained a significant risk factor for PE. In contrast, although patients with PE were older than those without in pooled results, the difference in age between the PE and non-PE groups became nonsignificant when one of 11 studies was omitted at a time^20,21,23–25,27–29,31,33,34^ or when studies of low quality were removed, indicating only a weak association between age and the risk of PE. Seven studies involving a total of 1925 patients with COVID-19 (PE group, n = 266 vs. non-PE group, n = 1659) were eligible for analysis on the association between BMI and risk of PE (Fig. 5C)^20,21,25–27,29,31^. There was no difference in BMI between PE and non-PE patients (MD = − 0.76 kg/m^2^, 95% CI − 1.78 to 0.25, *p* = 0.14; I^2^ = 28%) (Fig. 5C). The overall results of the impact of BMI on the risk of PE remained unchanged by removing either one study at a time or those of low quality. In addition, analysis of data from high-quality studies demonstrated that other comorbidities were non-significant risk factors for PE in the recruited COVID-19 patients (Table 3). Sensitivity analysis through omitting one study at a time showed no significant influence on the overall outcome.

**Table 3:** Calculated heterogeneity and effect size of potential risk factors for pulmonary embolism*

Comorbidity	No. of studies (n)	Pooled patients	I^2^ (%)	Odds ratio (95% CI)	*P* value
Heart failure	8	1989	0	1.15 (0.68–1.92)	0.6
Hypertension	9	2065	16	0.91 (0.67–1.24)	0.56
Pulmonary disease	6	1639	20	0.9 (0.48–1.68)	0.74
Smoking	6	1588	0	0.66 (0.39–1.1)	0.11
Diabetes mellitus	9	2065	0	0.83 (0.6–1.14)	0.25
Obesity	5	385	0	1.02 (0.64–1.64)	0.93
Chronic kidney disease	5	1908	0	1.02 (0.6–1.75)	0.93
Cancer	10	2279	28	0.72 (0.4–1.27)	0.25
Previous venous thromboembolic history	9	2203	1	1.48 (0.97–2.26)	0.07

CI, confidence interval.

*All studies included were of high quality.

#### Laboratory profiles in COVID-19 patients with and without pulmonary embolism

**Figure 6:**
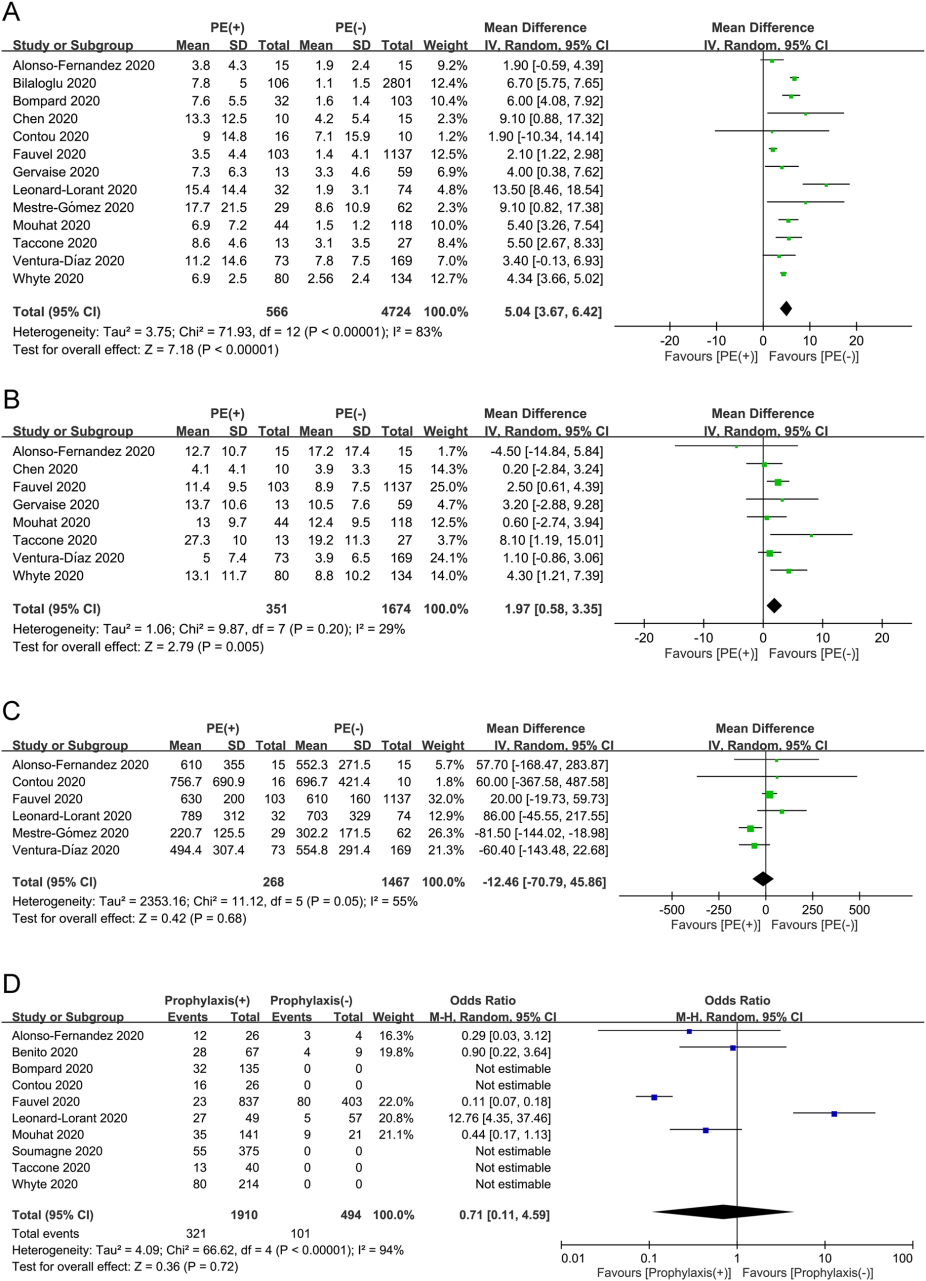
Forest plots for the difference in circulating concentrations of (**A**) D-dimer; (**B**) C-reactive protein; and (**C**) Fibrinogen between pulmonary embolism (PE) and non-PE groups; (**D**) Forest plot for comparing the risk of pulmonary embolism between patients with anticoagulant prophylaxis and those without. CI = confidence interval, IV = inverse variance, M–H = Mantel–Haenszel.

The laboratory profiles in COVID-19 patients with PE and in those without are demonstrated in Fig. 6A–C. There were 13, 8, and 6 studies available for D-dimer (PE group, n = 566 vs. non-PE group, n = 4724)^20,22–27,29–34^, C-reactive protein (CRP) (PE group, n = 351 vs. non-PE group, n = 1674)^20,24,26,27,31–34^, and fibrinogen (PE group, n = 268 vs. non-PE group, n = 1467)^20,25,26,29,30,33^analyses, respectively. The D-dimer (MD = 5.04 µg/mL, 95% CI 3.67 to 6.42, *p* < 0.00001, I^2^ = 83) (Fig. 6A) and CRP (MD = 1.97 mg/dL, 95% CI 0.58 to 3.35, *p* = 0.005, I^2^ = 29%) (Fig. 6B) concentrations were higher in PE patients compared with the levels in those without, while there was no difference in fibrinogen levels between PE and non-PE patients (MD = − 12.46 mg/dL, 95% CI − 70.79 to 45.86, *p* = 0.68, I^2^ = 55%) (Fig. 6C). Sensitivity analysis by removing one study at a time or those of low quality showed no significant impact on outcome by omitting certain trials. Funnel plot for D-dimer showed no apparent asymmetry (Fig. 4D), indicating a low risk of publication bias. Of the 16 included studies, 13 provided the circulating concentrations of D-dimer among COVID-19 patients with and without pulmonary embolism (Fig. 6A). The ranges of D-dimer concentration for those with and without PE were 3.5–17.7 μg/mL and 1.1–8.6 μg/mL, respectively. Of the 13 trials, 10 (76.9%) demonstrated a significantly higher circulating D-dimer level in patients with PE compared to that in the non-PE group (Fig. 6A).

#### The impact of anticoagulant prophylaxis on risk of pulmonary embolism

Ten studies involving a total of 2404 patients with COVID-19 (Prophylaxis group, n = 1910 vs. non-prophylaxis group, n = 494) were eligible for the analysis of the impact of anticoagulant prophylaxis on the risk of PE^20,21,23,25,26,29,31,32,34^. Forest plot demonstrated no significant impact of anticoagulant prophylaxis on the risk of PE in the recruited COVID-19 patients (OR = 0.71, 95% CI 0.11 to 4.59, *p* = 0.72; I^2^ = 94%) (Fig. 6D). However, sensitivity analysis showed that the use of anticoagulant prophylaxis was associated with a reduction in the risk of PE (OR = 0.31, 95% CI 0.1 to 0.91, *p* = 0.03, I^2^ = 75%) when the study by Leonard-Lorant et al.^29^ (i.e., a low-quality study) was omitted.

## Discussion

The present study represented the first meta-analysis investigating the mortality risk associated with pulmonary embolism (PE), the risk factors for PE, and the effectiveness of anticoagulant prophylaxis against PE in patients with COVID-19. The study addressed several important clinical issues. Contrary to previous findings of positive associations of PE with mortality, age, BMI, and other systemic diseases^4^, our results showed no significant correlations between PE and such comorbidities when compared with those in patients without PE. On the other hand, our results demonstrated that PE was significantly linked to the male gender as well as increased risks of mechanical ventilation and ICU admission. Besides, the current study demonstrated a positive correlation of PE with circulating D-dimer and CRP concentrations, which was consistent with the finding of previous studies^26,36,37^. Although some findings of the present meta-analysis were consistent with those previously reported, some of our results raised clinical issues that need to be addressed.

Previous review studies have shown a positive association between the severity of COVID-19 and the risk of thromboembolism^2,3^. The present meta-analysis further demonstrated that PE is a significant risk factor for mechanical ventilation and ICU admission for COVID-19 patients. Our findings were consistent with those of a previous multicenter cohort study that showed significantly higher rates of ICU transfer and mechanical ventilation in the PE group^26^. On the other hand, the lack of a significant association between the development of PE and mortality in the current study was contradictory to that in the non-COVID setting in which about 20% of patients with PE would die before diagnosis or shortly thereafter, especially for those with hemodynamic instability^38^. Albeit seemingly paradoxical, our finding may be attributed to the observation that despite the development of multiple thrombi in both medium- and small-sized blood vessels^39^ as well as fibrinous microthrombi in the arterioles of the lungs and other organs^40^, they may not be the direct cause of death^39,40^; an autopsy series on 21 COVID-19 patients attributed the primary cause of death to respiratory failure from exudative diffuse alveolar damage as well as massive capillary congestion with or without superimposed bronchopneumonia, while PE was noted in only four of the patients (i.e., less than one fifth)^41^. Besides, the wide adoption of anticoagulation prophylaxis against COVID-19-associated hypercoagulability in the included trials (11 out of 16) of the current study may have decreased the frequency of recurrent PE, which has been reported to be a major contributor to PE-associated mortality in the non-COVID-19 setting^38^. In addition, proactive survey programs for patients with COVID-19^6^ may limit the progression of PE and also the subsequent mortality.

Another interesting finding of the present study was the identification of the male gender as a significant risk factor for PE in COVID-19 patients. Although a previous multi-center cohort study has identified the male gender as a risk factor for PE in patients with COVID-19^26^, the published review studies did not reveal this finding^2,3^. Consistently, a large-scale study on patients without COVID-19 did not show an increased incidence of PE in males^38^. The disproportionately higher risk for males may be attributable to preexisting cardiovascular disease as well as COVID-19-associated cardiovascular injury^42^. On the other hand, although age is a known risk factor for PE in non-COVID patients^4,35^, our result showed no significant association between age and PE in those with COVID-19 on sensitivity analysis that demonstrated notable impacts from individual studies. Similarly, other systemic comorbidities known to be associated with PE in the non-COVID setting, including pulmonary diseases, obesity, heart failure, and cancer^4^ were also non-significant risk factors for PE.

Despite the lack of overall therapeutic benefit of anticoagulant prophylaxis against PE in the present meta-analysis, sensitivity analysis revealed a significant reduction in the risk of PE (OR = 0.31, 95% CI 0.1–0.91, *p* = 0.03, I^2^ = 75%) after omitting the study by Leonard-Lorant et al^29^. in which prophylactic anticoagulants were only given to COVID-19 patients highly suspected of having PE (e.g., elevated serum D-dimer concentration) instead of being a routine strategy. After confirming the diagnosis of PE through computed tomographic angiography, that study showed that 78% of patients in the PE group and 23% in the non-PE group received anticoagulant prophylaxis. Conceivably, that study may underestimate the benefit of anticoagulant prophylaxis as a routine treatment as reflected by the relatively low proportion of patients undergoing prophylaxis in the PE group, in which close to one-fourth (22%) did not receive prophylaxis because of the highly selective nature of the program. Consistently, a previous meta-analysis has demonstrated a positive association between a high rate of pharmacologic thromboprophylaxis (defined as ≥ 60%) and a lower incidence of thromboembolism^3^.

D-dimer, which is the degradation product of crosslinked fibrin (by factor XIII), reflects an ongoing activation of the hemostatic system and serves as an indicator of thrombosis^43^. The finding of a positive correlation between the circulating concentration of D-dimer and PE in the present study was consistent with that of previous studies on COVID-19 patients^36^. Nevertheless, there is no consensus on the optimal cut-off value and prognostic significance^2^. Although a previous report has demonstrated an association between a four-fold increase in circulating D-dimer concentration and mortality among COVID-19 patients^44^, we showed no significant correlation between PE and mortality even though the circulating D-dimer levels of COVID-19 patients with PE in the included studies were at least four-fold higher than that of normal (defined as < 0.4 μg/mL) (Fig. 6A). The lack of association between PE and mortality despite the elevated circulating D-dimer levels may be attributed to a short follow-up, the nature of PE as a peripheral disease with a relatively low disease severity, and timely detection with early intervention. In addition, although previous studies have identified an elevated circulating fibrinogen level as a risk factor for thromboembolism^6^, the present study demonstrated no significant relationship between fibrinogen concentration and PE in COVID-19 patients.

Focusing on CRP, a marker of systemic inflammation, the current study highlighted its positive association with PE. Our result was consistent with that of previous studies that identified vascular inflammation^9^ and an elevated CRP concentration^26,37^ as risk factors for PE in COVID-19 patients. Because CRP is more a marker of bacterial infections than that of viral diseases^45^, an elevated circulating CRP level may suggest secondary bacterial infections. Indeed, superimposing bacterial infection is known to contribute to mortalities and morbidities among patients with COVID-19^46^; a previous study reported an incidence of secondary bacterial infection in up to 41% of COVID-19 patients in critical condition^37^. Besides, a recent meta-analysis has identified procalcitonin, CRP, D-dimer, and lactate dehydogense as predictors of the severity of COVID-19 infections^47^. Consistently, our study demonstrated an elevated CRP level in COVID-19 patients with PE compared to that in those without. In terms of treatment, previous meta-analytical studies have shown a positive impact of immunological treatment by demonstrating the effectiveness of tocilizumab, a humanized monoclonal antibody clinically used as an immunosuppressive agent targeting the interleukin-6 receptor, for reducing the expressions of biomarkers^48^ and mortality rate^49^ in patients with COVID-19, underscoring the therapeutic potential of combining anti-cytokine and anticoagulant in patients with moderate to severe COVID-19 infections.

Our finding of significant positive associations of PE with the risks of mechanical ventilation and ICU admission but not mortality in COVID-19 patients may suggest an increased awareness among clinicians regarding the probability of PE to expedite the implementation of preemptive measures in this patient population. In addition, the lack of significant correlation between PE and systemic comorbidities previously reported to be related to PE (e.g., obesity) may highlight the need for suspecting PE in patients with COVID-19 even in the absence of conventional risk factors.

Moreover, although DVT is the major cause of PE in the non-COVID-19 setting^50^, our results demonstrated only a DVT prevalence of 11% in COVID-19 patients with PE. Therefore, instead of being dislodgement from a venous thrombus, our finding may implicate a different mechanism underlying clot formation in the pulmonary vasculature of patients with COVID-19 infection. The autopsy findings of diffuse alveolar damage with fibrinous microthrombi in the edematous and congested alveolar capillaries as well as evidence of damage to the airway surface epithelium^10^ appear to support this hypothesis.

Contrary to previous findings that supported the use of D-dimer level as a prognostic indicator for COVID-19 patients^36^, our results suggested that circulating D-dimer concentration could serve as a diagnostic clue for PE but not necessarily a prognostic indicator. Similarly, the lack of significant relationship between fibrinogen concentration and PE in COVID-19 patients in the current study may not support its use as a diagnostic tool for PE in this patient population. Furthermore, although the finding of an association between circulating CRP level and PE in the present study may suggest superimposing bacterial infection, evidence from autopsy of COVID-19 patients implicated the role of platelet activation rather than infectious pathogens in thrombosis formation^39^. The therapeutic potential of anti-platelet agents for prophylaxis against PE among COVID-19 patients remains to be elucidated.

One of the strengths of the present meta-analysis was our investigation into the risk factors and mortality as well as the risks of mechanical ventilation and ICU admission associated with PE in patients with COVID-19 instead of merely studying the incidence of PE and DVT. In addition to identifying unreported risk factors for PE in COVID-19 patients after analyzing the available clinical evidence, we found the lack of significant correlations between PE and a number of comorbidities previously proposed to be linked to PE development (i.e., smoking, obesity, chronic kidney disease, malignancy, and a previous history of venous thromboembolic disease). Furthermore, although a previous large-scale multicenter study on non-COVID-19 patients suggested a failure of anticoagulant prophylaxis against PE as one-third of patients were under prophylaxis at the time of PE occurrence^38^, our results support a beneficial role of prophylaxis in patients with COVID-19.

Nevertheless, the study had its limitations that need to be taken into consideration for accurate interpretation of its findings. First, for the purpose of the current study, only the highest value of the parameters was selected for analysis during a time course. The possibility that the data were acquired after confirmation of the diagnosis of PE by computed tomography pulmonary angiography (CTPA) may contribute to the wide range of variation in certain parameters (e.g., D-dimer). Besides, the difference in indications for CTPA (e.g., respiratory distress, elevated D-dimer levels, ICU admission status) may also affect the data for analysis. Second, despite the known ethnical impact on clinical outcomes among COVID-19 patients^51^, no information was available for evaluating a possible ethnical association with PE and its associated mortality in the current study. Third, the relatively short follow-up period in the majority of included studies (i.e., less than two months) may bias the outcomes. Fourth, because the optimal doses of anticoagulant for prophylaxis against PE remain unclear^2^, there were discrepancies in anticoagulant dosages in the included studies. Nevertheless, we demonstrated effectiveness of anticoagulant prophylaxis after exclusion of one study^29^. Fifth, although the presence of right ventricular hypokinesis has been reported to double the risk of mortality within three months of the PE diagnosis^38^, only a limited number of studies included in the present meta-analysis (7 out of 16) provided the information (Supplemental Table 3). Sixth, despite the potential confounding effects of thrombophilia, we were unable to perform a subgroup analysis because none of the included studies delineated this entity. Nevertheless, we demonstrated that the underlying conditions of the patients that may contribute to thrombophilia (i.e., smoking, obesity, chronic kidney disease, malignancy, and a previous history of venous thromboembolic disease)^52^ had no significant impact on the risk of PE. Finally, the heterogeneity of the recruited patients (e.g., inpatients vs. outpatients) as well as the severity of their diseases may impact the study outcomes.

## Conclusions

Through systematically reviewing the eligible clinical trials, the present meta-analysis demonstrated significant associations of pulmonary embolism with the male gender, risks of mechanical ventilation and ICU admission as well as elevated circulating concentrations of D-dimer and CRP in COVID-19 patients despite the lack of correlation between pulmonary embolism and mortality. After omitting one trial with strict patient selection criteria for anticoagulant prophylaxis, our results showed significant therapeutic benefit of anticoagulant prophylaxis against pulmonary embolism in those with COVID-19 infection. Because of limited data from the included observational studies, further large-scale clinical trials are warranted to support our findings.

## Supplementary Information

Supplementary Tables.

## References

[1] Raskob GE, Angchaisuksiri P, Blanco AN (2014). Thrombosis: A major contributor to global disease burden. Arterioscler. Thromb. Vasc. Biol..

[2] Al-Ani F, Chehade S, Lazo-Langner A (2020). Thrombosis risk associated with COVID-19 infection. A scoping review. Thromb. Res..

[3] Zhang C, Shen L, Le KJ (2020). Incidence of venous thromboembolism in hospitalized coronavirus disease 2019 patients: A systematic review and meta-analysis. Front. Cardiovasc. Med..

[4] Kahn SR, Comerota AJ, Cushman M (2014). The postthrombotic syndrome: Evidence-based prevention, diagnosis, and treatment strategies: A scientific statement from the American Heart Association. Circulation.

[5] Wichmann D, Sperhake JP, Lugehetmann M (2020). Autopsy findings and venous thromboembolism in patients with COVID-19. Ann. Intern. Med..

[6] Susen S, Tacquard CA, Godon A (2020). Prevention of thrombotic risk in hospitalized patients with COVID-19 and hemostasis monitoring. Crit. Care.

[7] Tang N, Bai H, Chen X, Gong JL, Li DJ, Sun ZY (2020). Anticoagulant treatment is associated with decreased mortality in severe coronavirus disease 2019 patients with coagulopathy. J. Thromb. Haemost..

[8] Trimaille A, Curtiaud A, Marchandot B (2020). Venous thromboembolism in non-critically ill patients with COVID-19 infection. Thromb. Res..

[9] Vinayagam S, Sattu K (2020). SARS-CoV-2 and coagulation disorders in different organs. Life Sci..

[10] Maiese A, Manetti AC, La Russa R (2020). Autopsy findings in COVID-19-related deaths: A literature review. Forensic Sci. Med. Pathol..

[11] Coccheri S (2020). COVID-19: The crucial role of blood coagulation and fibrinolysis. Intern. Emerg. Med..

[12] O'Donnell JS, Peyvandi F, Martin-Loeches I (2021). Pulmonary immuno-thrombosis in COVID-19 ARDS pathogenesis. Intensive Care Med..

[13] Townsend L, Fogarty H, Dyer A (2021). Prolonged elevation of D-dimer levels in convalescent COVID-19 patients is independent of the acute phase response. J. Thromb. Haemost..

[14] White D, MacDonald S, Bull T (2020). Heparin resistance in COVID-19 patients in the intensive care unit. J. Thromb. Thrombolysis.

[15] Tritschler T (2020). Anticoagulant interventions in hospitalized patients with COVID-19: A scoping review of randomized controlled trials and call for international collaboration. J. Thromb. Haemost..

[16] Artifoni M, Danic G, Gautier G (2020). Systematic assessment of venous thromboembolism in COVID-19 patients receiving thromboprophylaxis: Incidence and role of D-dimer as predictive factors. J. Thromb. Thrombolysis.

[17] Moher D, Liberati A, Tetzlaff J, Altman DG (2009). Preferred reporting items for systematic reviews and meta-analyses: the PRISMA statement. BMJ.

[18] Landis JR, Koch GG (1977). The measurement of observer agreement for categorical data. Biometrics.

[19] Wells, G. A. *et al.* The Newcastle-Ottawa Scale (NOS) for assessing the quality of nonrandomised studies in meta-analyses. http://www.ohri.ca/programs/clinical_epidemiology/oxford.asp (2014).

[20] Alonso-Fernandez A, Toledo-Pons N, Cosio BG (2020). Prevalence of pulmonary embolism in patients with COVID-19 pneumonia and high D-dimer values: A prospective study. PLoS ONE.

[21] Benito N, Filella D, Mateo J (2020). Pulmonary thrombosis or embolism in a large cohort of hospitalized patients with covid-19. Front. Med. (Lausanne).

[22] Bilaloglu S, Aphinyanaphongs Y, Jones S, Iturrate E, Hochman J, Berger JS (2020). Thrombosis in hospitalized patients with COVID-19 in a New York City Health System. JAMA.

[23] Bompard F, Monnier H, Saab I (2020). Pulmonary embolism in patients with COVID-19 pneumonia. Eur. Respir. J..

[24] Chen J, Wang X, Zhang S (2020). Characteristics of acute pulmonary embolism in patients with COVID-19 associated pneumonia from the City of Wuhan. Clin. Appl. Thromb. Hemost..

[25] Contou D, Pajot O, Cally R (2020). Pulmonary embolism or thrombosis in ARDS COVID-19 patients: A French monocenter retrospective study. PLoS ONE.

[26] Fauvel C, Weizman O, Trimaille A (2020). Pulmonary embolism in COVID-19 patients: A French multicentre cohort study. Eur. Heart J..

[27] Gervaise A, Bouzad C, Peroux E, Helissey C (2020). Acute pulmonary embolism in non-hospitalized COVID-19 patients referred to CTPA by emergency department. Eur. Radiol..

[28] Grillet F, Busse-Coté A, Calame P, Behr J, Delabrousse E, Aubry S (2020). COVID-19 pneumonia: Microvascular disease revealed on pulmonary dual-energy computed tomography angiography. Quant. Imaging Med. Surg..

[29] Léonard-Lorant I, Delabranche X, Séverac F (2020). Acute pulmonary embolism in patients with COVID-19 at CT angiography and relationship to d-Dimer levels. Radiology.

[30] Mestre-Gómez B (2020). Incidence of pulmonary embolism in non-critically ill COVID-19 patients. Predicting factors for a challenging diagnosis. J. Thromb. Thrombolysis.

[31] Mouhat B (2020). Elevated D-dimers and lack of anticoagulation predict PE in severe COVID-19 patients. Eur. Respir. J..

[32] Taccone FS (2020). Higher intensity thromboprophylaxis regimens and pulmonary embolism in critically ill coronavirus disease 2019 patients. Crit. Care Med..

[33] Ventura-Díaz S (2020). A higher D-dimer threshold for predicting pulmonary embolism in patients with COVID-19: A retrospective study. Emerg. Radiol..

[34] Whyte MB, Kelly PA, Gonzalez E, Arya R, Roberts LN (2020). Pulmonary embolism in hospitalised patients with COVID-19. Thromb. Res..

[35] Di Nisio M, van Es N, Buller HR (2016). Deep vein thrombosis and pulmonary embolism. Lancet.

[36] Yao Y, Cao J, Wang Q (2020). D-dimer as a biomarker for disease severity and mortality in COVID-19 patients: A case control study. J. Intensive Care.

[37] Keske S, Tekin S, Sait B (2020). Appropriate use of tocilizumab in COVID-19 infection. Int. J. Infect. Dis..

[38] Goldhaber SZ, Visani L, De Rosa M (1999). Acute pulmonary embolism: Clinical outcomes in the International Cooperative Pulmonary Embolism Registry (ICOPER). Lancet.

[39] Ghimire A, Subedi A, Bhattarai B, Sah BP (2020). The effect of intraoperative lidocaine infusion on opioid consumption and pain after totally extraperitoneal laparoscopic inguinal hernioplasty: A randomized controlled trial. BMC Anesthesiol..

[40] Nishiga M, Wang DW, Han Y, Lewis DB, Wu JC (2020). COVID-19 and cardiovascular disease: From basic mechanisms to clinical perspectives. Nat. Rev. Cardiol..

[41] Menter T, Haslbauer JD, Nienhold R (2020). Postmortem examination of COVID-19 patients reveals diffuse alveolar damage with severe capillary congestion and variegated findings in lungs and other organs suggesting vascular dysfunction. Histopathology.

[42] Medzikovic L, Cunningham CM, Li M (2020). Sex differences underlying preexisting cardiovascular disease and cardiovascular injury in COVID-19. J. Mol. Cell. Cardiol..

[43] Adam SS, Key NS, Greenberg CS (2009). D-dimer antigen: Current concepts and future prospects. Blood.

[44] Velavan TP, Meyer CG (2020). Mild versus severe COVID-19: Laboratory markers. Int. J. Infect. Dis..

[45] Korppi M, Kroger L (1993). C-reactive protein in viral and bacterial respiratory infection in children. Scand. J. Infect. Dis..

[46] Li X, Wang L, Yan S (2020). Clinical characteristics of 25 death cases with COVID-19: A retrospective review of medical records in a single medical center, Wuhan, China. Int. J. Infect. Dis..

[47] Hariyanto TI, Japar KV, Kwenandar F (2021). Inflammatory and hematologic markers as predictors of severe outcomes in COVID-19 infection: A systematic review and meta-analysis. Am. J. Emerg. Med..

[48] Hariyanto TI, Kurniawan A (2020). Tocilizumab administration is associated with the reduction in biomarkers of coronavirus disease 2019 infection. J. Med. Virol..

[49] Hariyanto TI, Hardyson W, Kurniawan A (2021). Efficacy and safety of tocilizumab for coronavirus disease 2019 (Covid-19) patients: A systematic review and meta-analysis. Drug Res. (Stuttg).

[50] Huisman MV, Barco S, Cannegieter SC (2018). Pulmonary embolism. Nat. Rev. Dis. Primers.

[51] Webb Hooper M, Napoles AM, Perez-Stable EJ (2020). COVID-19 and racial/ethnic disparities. JAMA.

[52] Campello E, Spiezia L, Adamo A, Simioni P (2019). Thrombophilia, risk factors and prevention. Expert Rev. Hematol..

