# Supplementary material for: Mortality and risk factors associated with pulmonary embolism in coronavirus disease 2019 patients: a systematic review and meta-analysis
Source: Sci Rep. 2021 Aug 6;11:16025. doi: 10.1038/s41598-021-95512-7 (PMC8346591; doi:10.1038/s41598-021-95512-7)
Supplement: Supplementary file 1 — Supplementary Tables. [file 41598_2021_95512_MOESM1_ESM.docx]

**Mortality and Risk Factors Associated with Pulmonary Embolism in Coronavirus Disease 2019 Patients: A Systematic Review and Meta-Analysis**

*Running head:* COVID-19 and pulmonary embolism

Carlos Andrés Gómez, BSc ^1,2^; Cheuk-Kwan Sun, MD, PhD ^1,3,^*****; I-Ting Tsai, MD^1,3^; Yang-Pei Chang^4,5^, MD; Ming-Chung Lin, MD^6^; I-Yin Hung, MD^6^; Ying-Jen Chang, MD^6^; Li-Kai Wang, MD^6^; Yao-Tsung Lin, MD^6^; Kuo-Chuan Hung, MD^6^

^1^ School of Medicine for International Students, College of Medicine, I-Shou University, Kaohsiung, Taiwan

^2^ Universidad Nacional Autónoma de Honduras en el Valle de Sula

^3^ Department of Emergency Medicine, E-Da Hospital, Kaohsiung, Taiwan

^4^ Department of Neurology, Kaohsiung Municipal Ta-Tung Hospital, Kaohsiung Medical University, Kaohsiung, Taiwan

^5^ Department of Neurology, Kaohsiung Medical University Hospital, Kaohsiung Medical University, Kaohsiung, Taiwan

^6^Department of Anesthesiology, Chi Mei Medical Center, Tainan, Taiwan

(*** signifies equal contribution compared to the first author**)

**Corresponding author:**

Kuo-Chuan Hung, MD

Department of Anesthesiology, Chi Mei Medical Center, Tainan, Taiwan

Chi Mei Medical Center, No.901, ChungHwa Road, YungKung Dist, Tainan 71004, Taiwan

E-mail: ed102605@gmail.com

TEL: +886-6-281-2811

FAX: +886-6-283-3806

**Supplemental Table 1. Search Strategies**

| **Database** | # | **Search syntax** |
| --- | --- | --- |
| **PubMed** | 1 | ("severe acute respiratory syndrome" or "coronavirus 2" or “coronavirus" or "corona virus" or "covid-19" or "nCoV" or "2019nCoV" or "Wuhan virus") |
|  | 2 | ("COVID-19" or "SARS-CoV-2") [MeSH Terms] |
|  | 3 | ("thromboembolism" or "clot" or "deep vein thrombosis" or "DVT" or "venous thromboembolism" or "pulmonary embolism" or "thrombosis" or "venous thrombosis") |
|  | 4 | "pulmonary embolism"[MeSH Terms] |
|  | 5 | (1 OR 2) AND (3 OR 4) |
| **Embase** | 1 | ("severe acute respiratory syndrome" or "coronavirus 2" or “coronavirus" or "corona virus" or "covid-19" or "nCoV" or "2019nCoV" or "Wuhan virus"):ti,ab,kw,de |
|  | 2 | "coronavirus disease 2019"/exp [Emtree Terms] |
|  | 3 | ("thromboembolism" or "clot" or "deep vein thrombosis" or "DVT" or "venous thromboembolism" or "pulmonary embolism" or "thrombosis" or "venous thrombosis"):ti,ab,kw,de |
|  | 4 | "lung embolism"/exp [Emtree Terms] |
|  | 5 | (1 OR 2) AND (3 OR 4) |
| **Cochrane CENTRAL** | 1 | ("severe acute respiratory syndrome" or "coronavirus 2" or “coronavirus" or "corona virus" or "covid-19" or "nCoV" or "2019nCoV" or "Wuhan virus"):ti,ab,kw |
|  | 2 | [mh " COVID-19"] or [mh " SARS-CoV-2"] [MeSH Terms] |
|  | 3 | ("thromboembolism" or "clot" or "deep vein thrombosis" or "DVT" or "venous thromboembolism" or "pulmonary embolism" or "thrombosis" or "venous thrombosis"):ti,ab,kw |
|  | 4 | [mh "pulmonary embolism"] [MeSH Terms] |
|  | 5 | (#1 OR #2) AND (#3 OR #4) |
| **Web of science** | 1 | TI=("severe acute respiratory syndrome" or "coronavirus 2" or “coronavirus" or "corona virus" or "covid-19" or "nCoV" or "2019nCoV" or "Wuhan virus") or AB=("severe acute respiratory syndrome" or "coronavirus 2" or “coronavirus" or "corona virus" or "covid-19" or "nCoV" or "2019nCoV" or "Wuhan virus") or KP=("severe acute respiratory syndrome" or "coronavirus 2" or “coronavirus" or "corona virus" or "covid-19" or "nCoV" or "2019nCoV" or "Wuhan virus") |
|  | 2 | (TI=("thromboembolism" or "clot" or "deep vein thrombosis" or "DVT" or "venous thromboembolism" or "pulmonary embolism" or "thrombosis" or "venous thrombosis") or AB=("thromboembolism" or "clot" or "deep vein thrombosis" or "DVT" or "venous thromboembolism" or "pulmonary embolism" or "thrombosis" or "venous thrombosis") or KP=("thromboembolism" or "clot" or "deep vein thrombosis" or "DVT" or "venous thromboembolism" or "pulmonary embolism" or "thrombosis" or "venous thrombosis")) |
|  | 3 | 1 AND 2 |

**Supplemental Table 2.** Indications for computed tomography pulmonary angiography

| Study | Screening criteria for PE |
| --- | --- |
| Alonso-Fernandez 2020 | At least one D-dimer value higher than 1 μg/mL during hospitalization |
| Benito 2020 | Based on clinical symptom and sign (e.g., persistently high D-dimer (>3,000 ng/mL) and/or hemodynamic deterioration or other “classic” symptoms of PE) |
| Bilaloglu 2020 | No standardized criteria, based on routine clinical care |
| Bompard 2020 | NA |
| Chen 2020 | Patients with an elevated D-dimer level or accompanying symptom(s), including chest pain, hemoptysis, and dyspnea. |
| Contou 2020 | Sudden circulatory or/and respiratory worsening with no obvious explanation |
| Fauvel 2020 | Supplementary oxygen was needed in COVID-19 patients with limited disease extension, or when unenhanced CT findings could not explain the severity of respiratory failure |
| Gervaise 2020 | Worsening of the patient’s clinical condition with new onset of dyspnea, desaturation, or chest pain and also an increase in D-dimer levels |
| Grillet 2020 | Severe grade infection were present, need for invasive mechanical ventilation, or when the patient suffered from comorbidities of active neoplasia, immunosuppression, history of organ or bone-marrow transplantation. |
| Leonard-Lorant 2020 | Suspicion of PE or other CT indication |
| Mestre-Gómez 2020 | Respiratory deterioration not attributable to other causes, data on acute respiratory distress without improvement despite specific treatment or elevation of D-dimer in discordance with other inflammatory parameters. |
| Mouhat 2020 | Respiratory distress or rapid clinical worsening |
| Soumagne 2020 | NA |
| Taccone 2020 | NA |
| Ventura-Díaz 2020 | Clinical suspicion of PE |
| Whyte 2020 | Clinical suspicion of PE based on Wells rule and level of D-dimer |

PE, pulmonary embolism; NA, not available; COVID-19, coronavirus disease 2019; CT, computed tomography;

**Supplemental Table 3.** Detection of deep vein thrombosis after computed tomography pulmonary angiography

| Study | Number of  patients with PE | Description | Incidence of PE and DVT |
| --- | --- | --- | --- |
| Alonso-Fernandez 2020 | 15 | NA | NA |
| Benito 2020 | 32 | Two PE patients had DVT | 6.3% |
| Bilaloglu 2020 | 106 | NA | NA |
| Bompard 2020 | 32 | NA | NA |
| Chen 2020 | 10 | No DVT in PE patients | 0% |
| Contou 2020 | 16 | Three PE patients had DVT | 19% |
| Fauvel 2020 | 103 | Twelve PE patients had lower extremity DVT | 11.7% |
| Gervaise 2020 | 13 | NA | NA |
| Grillet 2020 | 29 | NA | NA |
| Leonard-Lorant 2020 | 32 | NA | NA |
| Mestre-Gómez 2020 | 29 | NA | NA |
| Mouhat 2020 | 44 | NA | NA |
| Soumagne 2020 | 55 | Eleven PE patients had DVT | 20% |
| Taccone 2020 | 13 | Lower limb echo-Doppler was performed in 11 PE patients and revealed no DVT | NA |
| Ventura-Díaz 2020 | 73 | Five PE-positive patients had DVT | 6.8% |
| Whyte 2020 | 80 | Lower limb venous ultrasonography was performed in 19 patients; DVT was confirmed  in 11 patients (n = 7 with confirmed PE). | NA |

PE, pulmonary embolism; NA, not available; DVT, deep vein thrombosis.

**Supplemental Table 4.** Location of embolus and incidence of right ventricle dysfunction

| Study | Number of  patients with PE | Location of embolus  (segmental artery and subsegmental artery) | Incidence of RVD |
| --- | --- | --- | --- |
| Alonso-Fernandez 2020 | 15 | 60% | NA |
| Benito 2020 | 32 | 78.1% | 15.6% |
| Bilaloglu 2020 | 106 | NA | NA |
| Bompard 2020 | 32 | 68.8% | 18.8% |
| Chen 2020 | 10 | 100% | NA |
| Contou 2020 | 16 | 63% | NA |
| Fauvel 2020 | 103 | NA | NA |
| Gervaise 2020 | 13 | 55% | 38% |
| Grillet 2020 | 29 | 83% | NA |
| Leonard-Lorant 2020 | 32 | 44% | NA |
| Mestre-Gómez 2020 | 29 | 68.9% | NA |
| Mouhat 2020 | 44 | 56.8% | 15.9% |
| Soumagne 2020 | 55 | NA | NA |
| Taccone 2020 | 13 | 84.6% | 15.4% |
| Ventura-Díaz 2020 | 73 | 63% | 20.5% |
| Whyte 2020 | 80 | 51.25% | 11.3% |

RVD, right ventricular dysfunction (defined as right ventricular-to-left ventricular [RV/LV] ratio ≥1); NA, not available;
